# GOLD COPD DOCUMENT 2023: a brief update for practicing cardiologists

**DOI:** 10.1007/s00392-023-02217-0

**Published:** 2023-05-26

**Authors:** Alvar Agusti, Michael Böhm, Bartolomé Celli, Gerard J. Criner, Ana Garcia-Alvarez, Fernando Martinez, Don D. Sin, Claus F. Vogelmeier

**Affiliations:** 1 Hospital Clinic, IDIBAPS, University of Barcelona, Barcelona, Spain; 2 KardiologieAngiologie und Internistische Intensivmedizin, Universitätsklinikum des SaarlandesKlinik für Innere Medizin III, Saarland University, Homburg/Saar, Germany; 3 Brigham and Women’s Hospital, Harvard Medical School, Boston, USA; 4 Department of Thoracic Medicine and Surgery at the Lewis Katz School of Medicine, Philadelphia, PA USA; 5 Weill Cornell Medicine, NYPresbyterian Hospital, New York, NY USA; 6 Centre for Heart Lung Innovation, Department of Medicine (Division of Respirology), St. Paul’s Hospital, University of British Columbia, Vancouver, Canada; 7 Department of Medicine, Pulmonary and Critical Care Medicine, Member of the German Center for Lung Research (DZL), University of Marburg, Marburg, Germany

**Keywords:** Chronic bronchitis, Emphysema, GOLD, Smoking, Tobacco

## Abstract

Many patients seen by cardiologists suffer chronic obstructive pulmonary disease (COPD) in addition to their primary cardiovascular problem. Yet, quite often COPD has not been diagnosed and, consequently, patients have not been treated of their pulmonary disease. Recognizing and treating COPD in patients with CVDs is important because optimal treatment of the COPD carries important benefits on cardiovascular outcomes. The Global Initiative for Chronic Obstructive Lung Disease (GOLD) publishes an annual report that serves as a clinical guideline for the diagnosis and management of COPD around the world and has very recently released the 2023 annual report. Here, we provide a summary of the GOLD 2023 recommendations that highlights those aspects of more interest for practicing cardiologists dealing with patients with CVD who may suffer COPD.

## Introduction

Many patients seen by cardiologists suffer chronic obstructive pulmonary disease (COPD) in addition to their primary cardiovascular problem [1]. Yet, quite often, COPD has not been diagnosed and, consequently, patients have not been treated [1]. Recognizing and treating COPD in patients with CVDs are important because optimal treatment of the COPD carries important benefits on cardiovascular outcomes [2].

The Global Initiative for Chronic Obstructive Lung Disease (GOLD) publishes an annual report that serves as a clinical guideline for the diagnosis and management of COPD around the world and has very recently released the 2023 annual report [2]. Here, we provide a summary of the GOLD 2023 recommendations that highlights those aspects of more interest for practicing cardiologists dealing with patients with CVD who may suffer COPD [2]. It does not cover in detail all aspects of COPD. We refer the interested reader to the full GOLD document, the pocket guide, the slide deck, and/or the GOLD App, all freely downloadable from the GOLD website (www.goldcopd.org). Likewise, we acknowledge that other recent, global reviews in COPD may also be informative [3].

## Definition and burden

GOLD 2023 now defines COPD as a “heterogeneous lung condition characterized by chronic respiratory symptoms (dyspnea, cough, expectoration, exacerbations) due to abnormalities of the airways (bronchitis, bronchiolitis) and/or alveoli (emphysema) that cause persistent, often progressive, airflow obstruction” [4]. COPD may be punctuated by periods of acute worsening of respiratory symptoms, called exacerbations (ECOPD), which can be prevented and may require specific treatment and, sometimes, hospitalization [5]. In many patients, COPD co-exist with other significant concomitant chronic diseases, particularly CVD, such as coronary artery disease (CAD) and heart failure (HF), as well as diabetes, osteoporosis, cancer, and skeletal muscle dysfunction, among others [2].

The global prevalence of COPD oscillates around 12% of the general population. COPD currently ranks as the 3rd cause of mortality in the world (responsible for 3 million deaths), just behind CVDs [6]. The incidence of COPD is expected to rise over the next 40 years. By 2060, it is estimated that there may be over 5.4 million deaths annually from COPD [7, 8]. COPD prevalence and mortality are similar in males and females [9]. COPD has a significant economic burden; in the European Union, the total annual direct costs of COPD are estimated to be about 38.6 billion Euros [10].

## Causes of COPD: beyond tobacco smoking

COPD has been traditionally understood as a *self-inflicted* disease caused by an abnormal inflammatory response elicited by *tobacco smoking* occurring in *older males* [11]. Tobacco smoking certainly is a major environmental risk factor for COPD, but it is *not the only one* since other environmental exposures, such as biomass fuel, air pollution, and occupational particles, can also cause COPD [2]. In fact, between 20 and 40% of COPD patients worldwide are never smokers [12].

Genetic abnormalities can predispose individuals to COPD. The best documented genetic risk factor for COPD is α1 antitrypsin deficiency (caused by a mutation in the SERPINA1 gene), although it occurs in a minority (0.12%) of COPD patients, more in Northern Europe [13]. As *per* today, almost 100 single-nucleotide polymorphisms have been associated with increased risk of COPD, but their individual effect size is small, so COPD is now considered a polygenic disease [14].

Recent research has shown that abnormal lung development before and after birth due to factors like maternal smoking, prematurity, low birthweight, repeated infections in infancy, and/or poor nutrition, among others, can lead to COPD in young adults [15, 16]. Importantly, these young individuals show a higher prevalence and earlier incidence of comorbidities (e.g., CVD and diabetes) and die prematurely [17].

## Diagnosis of COPD

**Fig. 1:**
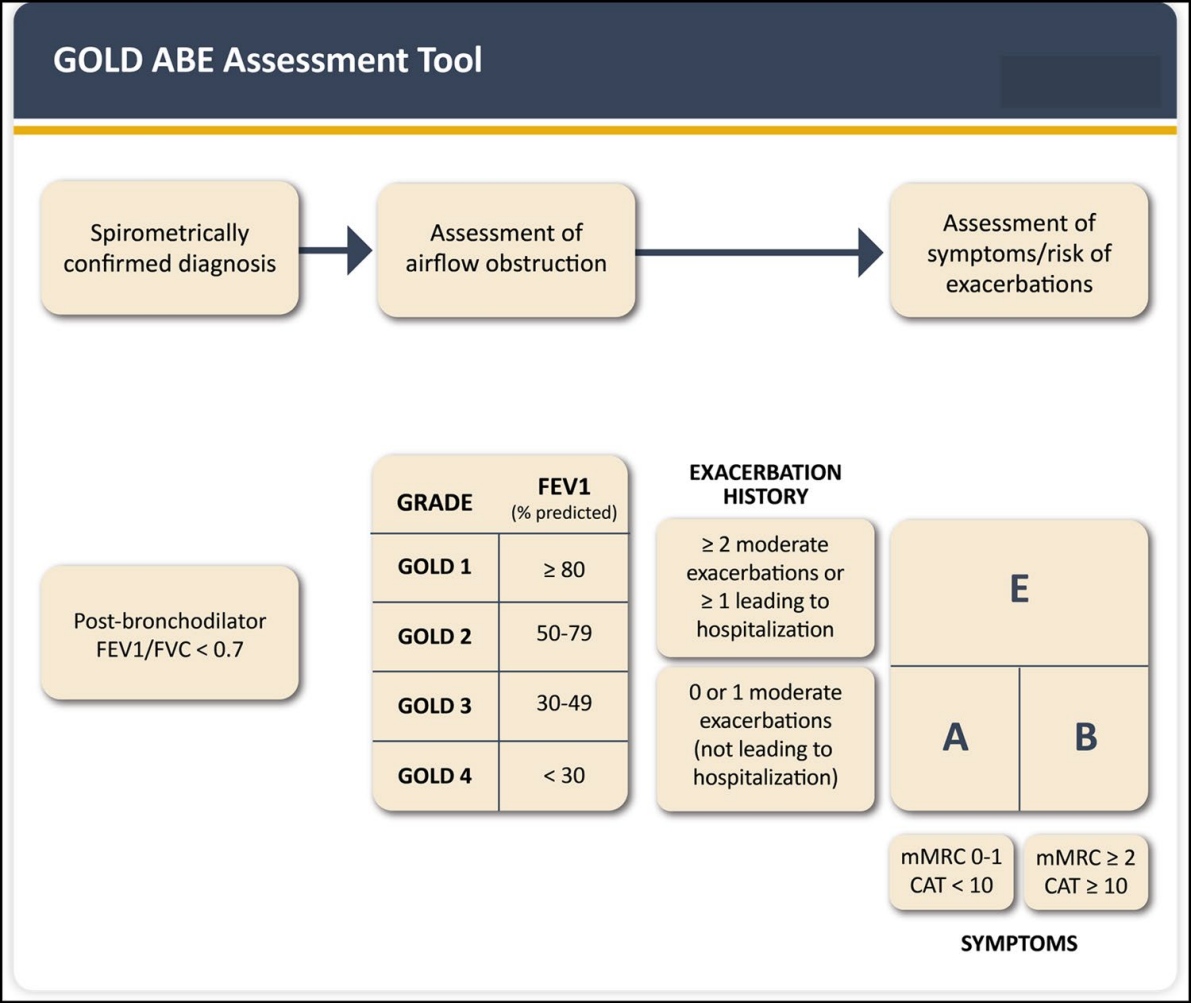
Diagnosis, assessment of the severity of airflow obstruction, and proposed ABE clinical assessment tool for COPD patients. Reproduced with permission from Ref. [2]

Under-diagnosis of COPD in the general population is huge, in the range of 70–80%, so the vast majority of COPD patients in the community are not treated at all [2]. COPD should be suspected in any patient who has dyspnea on exercise, chronic cough or sputum production, and/or a history of exposure to risk factors for the disease (see above), a constellation of clinical symptoms quite frequent in patients with CVDs, particularly HF. However, spirometry is necessary to establish the diagnosis of COPD by showing persistent airflow limitation after bronchodilation [2]. The latter is identified by a ratio between the volume of gas expired in the first second (FEV1) and the total volume of gas expired (Forced Vital Capacity—FVC) < 0.7 [18] (Fig. 1). The severity of airflow obstruction is established based on the FEV1 value (GOLD grades—Fig. 1). Clinical assessment is based on the presence of symptoms (low vs. high) and the previous history of ECOPD (≤ 1 moderate ECOPD in the previous year vs. more than that or one severe (hospitalized) ECOPD). Using these two dimensions, GOLD 2023 proposes to classify COPD patients in one of three groups (A, B, or E) (Fig. 1).

Mucus hypersecretion, resulting in chronic productive cough (*chronic bronchitis*) is not necessarily associated with airflow limitation; conversely, not all patients with COPD have mucus hypersecretion [2]. In some patients with chronic asthma, a clear distinction from COPD may be difficult since the two conditions share common traits and clinical expressions. HF can mimic or coexist with COPD. Sometimes it may be difficult to discern whether dyspnea (or poor functional class) is a consequence of COPD and/or HF, especially in patients with preserved left ventricular systolic function as diastolic dysfunction and COPD frequently coexist [19, 20].

Physical examination is rarely diagnostic in COPD except in cases of advanced emphysema where hyper-inflated thorax and decreased breath sounds with hyper-resonance may suggest the presence of the disease. Chest X-ray is not helpful to establish a diagnosis in COPD albeit suggestive changes include signs of lung hyperinflation (flattened diaphragm and an increase in the volume of the retrosternal air space), hyper-lucency, and rapid tapering of the vascular markings. It may, however, help to exclude alternative diagnoses and may give indications for the presence of significant respiratory (pulmonary fibrosis, bronchiectasis, and pleural diseases), skeletal (e.g., kyphoscoliosis), and cardiac (e.g., cardiomegaly) comorbidities [2]. By contrast, computed tomography (CT) of the chest is becoming more and more relevant due to increasing availability, reduced radiation doses, and reduced costs [21]. CT can characterize changes in the lungs (such as airway width and walls, mucus plugs, bronchiectasis, and type, spatial distribution and extent of emphysema, interstitial abnormalities, nodules and lymph nodes, and size of the pulmonary arteries). Besides, coronary artery calcification, that may be of prognostic relevance in COPD patients [22], osteoporosis (reduced bone mineral density in the vertebrae), cachexia (diminished area of the pectoralis muscle), and lung tumors may also be identified by CT [2].

Besides spirometry, other lung function measurements may be informative in some patients. For instance, the measurement of lung volumes (gas trapping, lung hyperinflation) by body plethysmography may be relevant to select patients for interventional procedures (e.g., endobronchial valve placement in patients with hyperinflation due to emphysema). Other functional measurements like the diffusing capacity of the lungs for carbon monoxide (DLco) [23], pulse oximetry, and arterial blood gasses can contribute to better characterize the type and therapeutic needs (e.g., oxygen therapy) of some COPD patients [2]. Finally, objectively measured exercise impairment, assessed by a 6-min walk test or other tests, is a powerful indicator of health status impairment and predictor of prognosis, both in patients with COPD and CVDs, and laboratory exercise testing can help identify the mechanisms of dyspnea, exercise limitation, and co-existing or alternative comorbid conditions [2].

## Primary and secondary prevention

The following measures must be considered for primary (disease occurrence) and/or secondary (disease progression) prevention. Smoking avoidance/cessation is the single most important preventive intervention [2]. Counseling delivered by healthcare professionals, even if brief (3 min), improves quit rates, but nicotine replacement and pharmacotherapy reliably increase long-term smoking abstinence rates [2]. Yet, the following aspects need to be considered in patients with CVDs. Nicotine replacement products (nicotine gum, inhaler, nasal spray, transdermal patch, sublingual tablet, or lozenge) reliably increases long-term smoking abstinence rates [2], but medical contraindications to nicotine replacement therapy include recent myocardial infarction or stroke [24, 25]. The contraindication to nicotine replacement therapy soon after acute coronary syndrome remains unclear [26, 27]. On the other hand, varenicline and bupropion increase long-term quit rates, but should always be used as a component of a supportive intervention program rather than as a sole intervention for smoking cessation [2]. The effectiveness of the antihypertensive drug clonidine for smoking cessation is limited by side effects [2]. Electronic cigarettes (e-cigarettes, vaping) are not recommended as a smoking cessation aid [2].

Vaccination is key preventive measurement in COPD [2]: (1) Influenza vaccination can reduce serious illness and death in COPD patients [2] and can also decrease the risk of acute coronary events and other endpoints in patients with a recent cardiac event [28]; (2) Pneumococcal vaccine with conjugate vaccine (PCV13) or pneumococcal polysaccharide vaccine (PPSV23) is recommended for all patients ≥ 65 years of age [2]. The PPSV23 is also recommended for younger COPD patients with significant comorbid conditions including chronic heart disease [2]; the CDC recommends one dose of a 20-valent pneumococcal conjugate vaccine (PCV20); or one dose of a 15-valent pneumococcal conjugate vaccine followed by PPSV23. We would like to mention that PPSV23 is still recommended in most countries, but the new conjugate vaccines had not been part of the evaluation for these recommendations. However, this will change in the near future. (3) COVID-19 vaccination (including boosters) is recommended in all patients with COPD [2]; (4) Pertussis (whooping cough), tetanus, and diphtheria (Tdap) vaccine is recommended in those who were not vaccinated in adolescence; (5) Herpes zoster vaccine to protect against shingles is recommended in adults with COPD older than 50 years of age; and, finally, (6) new respiratory syncytial virus (RSV) vaccines are under development.

## Initial pharmacologic treatment

**Fig. 2:**
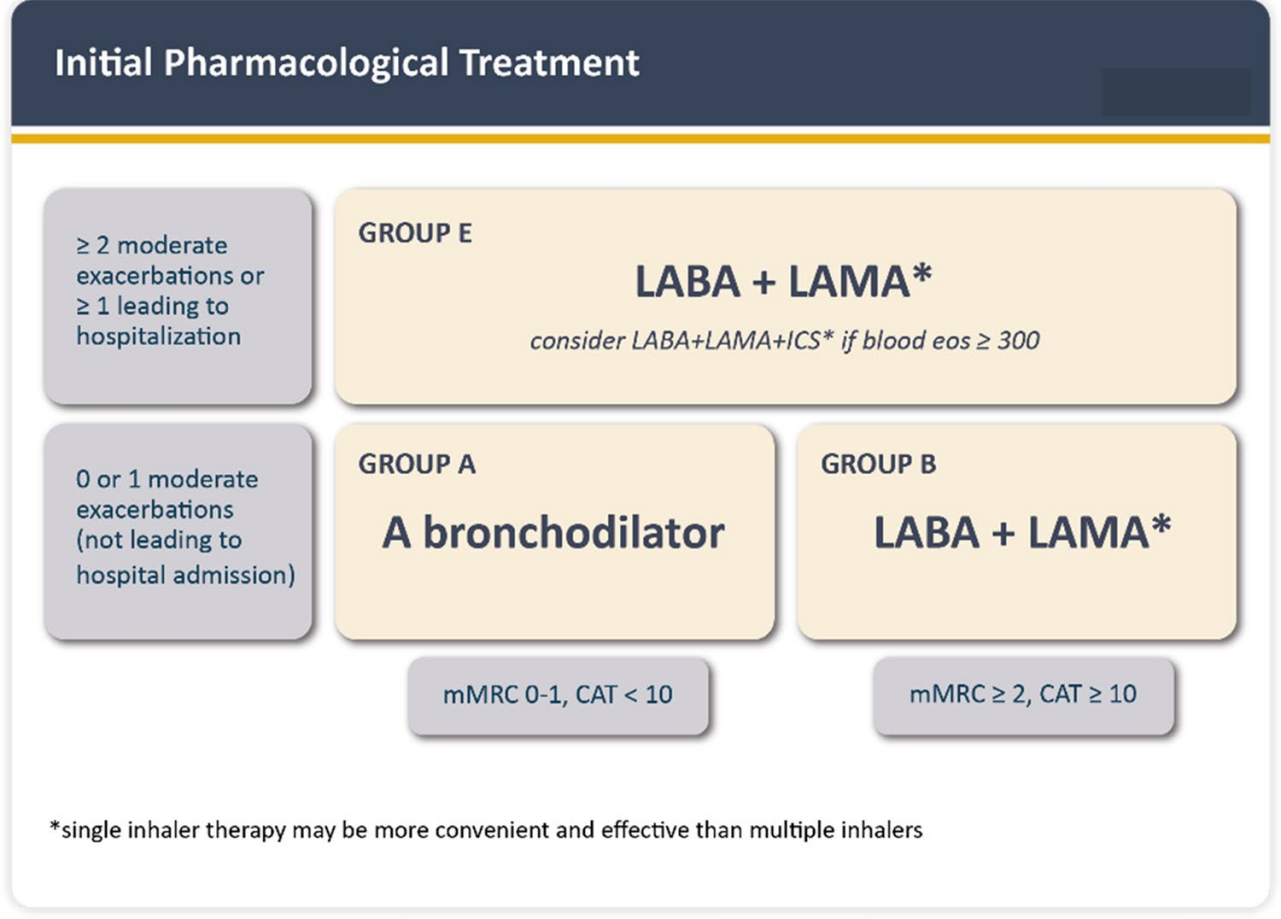
Recommended initial pharmacological treatment of COPD patients. To assess the level of symptoms, two simple and validated questionnaires are recommended: the modified Medical Research Council dyspnea scale (mMRC) with one question [67] or the COPD Assessment Test™ (CAT) with eight questions [68]. Abbreviations: eos: blood eosinophil count in cells per microliter. Reproduced with permission from Ref. [2]

Pharmacological therapy can reduce COPD symptoms, reduce the frequency and severity of exacerbations, and improve health status and exercise tolerance [2]. Recent evidence suggests beneficial effects on rates of lung function decline and mortality [2]. Here we only discuss the *initial pharmacological* treatment of the clinically *stable* patient with COPD (Fig. 2) because this likely is the most relevant aspect for a cardiologist treating patients with CVDs *and* COPD. We do not discuss treatment changes during follow-up or the treatment of the patient during an ECOPD episode [2], but we recommend that the practicing cardiologist refers the patient to a pulmonologist if initial treatment does not improve the health condition of the patient.

The cornerstone of COPD pharmacologic treatment is (in combination with quitting smoking) treatment with one (in patients with few symptoms) or two (most often) inhaled long-acting bronchodilators (LABD). If there are safety concerns for long-acting *ß*2 agonists (e.g., history of atrial fibrillation) or long-acting anticholinergics (e.g., severe glaucoma), monotherapy may be considered in patients with a symptom load that would normally lead to dual therapy. Short-acting bronchodilators, such as salbutamol (albuterol) or ipratropium, should be used only as a rescue medication. Short-acting *β*2 agonists are not recommended for regular chronic use in patients with arterial hypertension or heart failure. The decision on what is the most appropriate pharmacologic treatment alternative for a given patient requires consideration of both the inhaler device to use and the most appropriate molecule(s) for the patient.

### What inhaler device?

Not all inhaler devices (and the patients to use them) are equal. There are two main types of *portable* inhalers (we do not discuss here the role of nebulizers, which may be considered as home treatments in some patients): pressurized metered dose inhaler (pMDI) and dry powder inhalers (DPI). A pMDI delivers a specific (metered) dose of medication in the form of a short burst of aerosolized medicine utilizing the energy of compressed propellant(s) for the aerosol generation. pMDIs can deliver metered drug doses either directly (most patients) or via add-on holding chambers (in patients who are unable to synchronize dose release, inspiration, and breath-hold). Alternatively, DPIs deliver medication to the lungs in the form of a dry powder which can be held either in a capsule for manual loading or in a proprietary form inside the inhaler. Patients with severe airflow limitation may not be able to generate sufficient inspiratory flow to use a DPI efficiently; in these patients, pMDI (often via add-on plastic chambers) may be the preferred choice. A soft-mist inhaler, which does not use a propellant, is also a potential alternative. A proper inhalation technique is essential for the proper use of both pMDI and DPI (thus for treatment efficiency) and includes, in this order: (1) deep exhalation of thoracic gas; (2) deep and continuous inspiration (after the actuation of the device in the case of pMDI with or without chamber); and (3) breath holding for several seconds to allow proper deposition of the drug in the lungs. Proper inhalation technique should be checked periodically by the attending physician or an educated nurse [2].

### Which molecule(s)?

Pharmacological treatment should be individualized and guided by the severity of symptoms, risk of exacerbations, side effects, comorbidities, drug availability, cost and the patient’s response, preference, and ability to use various drug delivery devices.

There are two main pharmacological classes of LABD to use in patients with COPD: the *β*2 adrenergic agonists (LABA) and the muscarinic antagonists (LAMA). LABA include salmeterol, formoterol, indacaterol, vilanterol, and olodaterol. LAMA include tiotropium, aclidinium, umeclidinium, and glycopyrronium. Figure 2 presents the GOLD 2023 ABE quadrants used to guide initial pharmacological treatment in a patient with COPD. The three groups showed (A–E) results from the assessment of the level of symptoms/impairment experienced by the patient (low or high) in the horizontal axis and the history of ECOPD experienced in the previous year (low or high), which is the best predictor of future ECOPD episodes [2]. Although there are several potential recommendations depending on the assigned group, as a rule of thumb, the use of a LAMA/LABA combination achieves better results than the single components regarding lung function and symptoms irrespective of baseline impairment of health status. Therefore, LAMA/LABA combinations may be considered as initial maintenance therapy for symptomatic patients with COPD across a broad range of symptom severities [29]. These preparations can be prescribed once or twice daily depending on the commercial product and level of symptoms of the patient, once daily dosing better suiting patients with fewer symptoms and twice daily dosing preferred for those who are highly dyspneic. Chronic treatment with LABA–LAMA can be maintained in patients with CVDs, even in those treated with *β*-blockers.

A second group of drugs is represented by inhaled corticosteroids (ICS), of which there are several in the market (budesonide, beclomethasone, fluticasone propionate, and fluticasone furoate). In patients with COPD (asthma is different), they should never be administered as a single agent or in combination with LABA (LABA–ICS), but rather used in combination with LABA–LAMA (triple therapy), now available in a single canister, which is preferable to multiple canisters [2]. GOLD 2023 recommends considering *initial* treatment with triple therapy in E patients if their level of circulating eosinophils is higher than 300 cells/μL [2] (Fig. 2). Importantly, two recent, large randomized clinical trials have shown that triple therapy reduces all-cause mortality in GOLD E patients [30, 31].

**Fig. 3:**
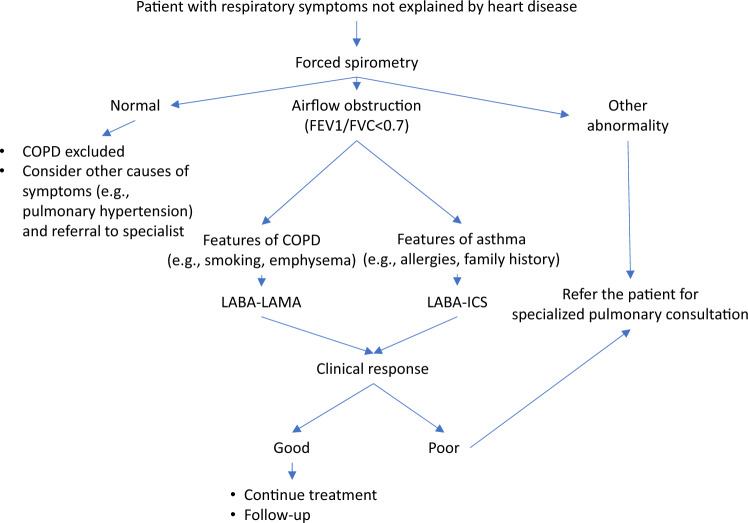
Proposed clinical strategy for a patient with respiratory symptoms seen by a cardiologist not explained by heart disease. For further explanations, see text

We recommend that cardiologists become familiar with several of the products available, so they become comfortable in initiating therapy in the event of absence of pulmonary specialized support at the moment of diagnosis, but whenever in doubt, a consultation with a pulmonary specialist is advised (Fig. 3).

## Non-pharmacological treatments

Non-pharmacological measures are important in COPD. Physicians should emphasize the importance of a healthy environment (avoiding both active and passive smoking, as well as air pollution, both inside and outside), encourage physical activity, and enroll the patient in a pulmonary rehabilitation program, particularly if the patient suffers from COPD plus a CVD, where the role of rehabilitation is well established [32, 33]. Vaccine use has been discussed above. If the patient remains symptomatic despite initial pharmacological and non-pharmacologic treatment, referral for a specialized assessment and management is encouraged.

## What about exacerbations?

GOLD 2023 has renewed the definition of an exacerbation of COPD (ECOPD) following the so-called Rome proposal [5]. Accordingly, an ECOPD is now defined as an event characterized by increased dyspnea and/or cough and sputum that worsens in < 14 days which may be accompanied by tachypnea and/or tachycardia and is often associated with increased local and systemic inflammation caused by infection, pollution, or other insult to the airways [5]. The time frame proposed (< 14 days) aims at differentiating ECOPD from progression of COPD, but many patients with ECOPD consult much earlier.

ECOPD are the major cause of morbidity and mortality in patients with COPD and account for 31–68% of COPD total care costs; hospitalizations account for most of these costs [34, 35]. Comorbid diseases frequently occurring in patients with (acute myocardial infarction, congestive heart failure, cardiac arrhythmias, and pulmonary embolism) can mimic or aggravate the symptoms of ECOPD and contribute to diagnostic confusion [36]. The reverse is also true [5]. A patient with HF and worsening dyspnea can actually be misdiagnosed of pulmonary congestion when he/she may suffer ECOPD. Appropriate investigations using electrocardiogram, echocardiogram, and biomarkers, such as N-terminal pro-brain natriuretic peptide (NT-pro-BNP) and troponin or d-dimers, and chest CT angiography are generally required to exclude acute cardiac events or pulmonary embolism, respectively, in these patients [37]. Treatment of ECOPD requires short-acting bronchodilators for relief of dyspnea, systemic corticosteroids, antibiotics if purulent sputum exists (where possible, sputum culture is recommended) and correction of hypoxemia with use of supplemental oxygen [18]. All these measures can be taken by a cardiologist, but if the exacerbation is severe, we would recommend referring the patient to an emergency department where non-invasive ventilation can be applied to correct hypercapnia and acidosis [2]. This therapeutic strategy can be used in patients with CVDs.

## Special considerations for cardiologists dealing with COPD patients

### Arterial hypertension

Arterial hypertension is the most frequently occurring comorbidity in COPD and may have implications for prognosis [38, 39]. Diastolic dysfunction as a result of sub-optimally treated hypertension may be associated with exercise intolerance and mimic symptoms associated with an acute exacerbation, thereby provoking hospitalization in COPD [40]. Thus, it is important to control blood pressure in COPD patients with underlying hypertension [41, 42]. Hypertension should be treated according to usual guidelines since there is no evidence that hypertension should be treated differently in the presence of COPD [2].

### Heart failure (HF)

The prevalence of systolic or diastolic HF in COPD patients ranges from 20 to 70% and its annual incidence between 3 and 4% [40]. Unrecognized HF may mimic or accompany COPD especially during ECOPD events [43]. Indeed, approximately 40% of COPD patients who are mechanically ventilated because of hypercapnic respiratory failure have evidence of left ventricular dysfunction [44, 45]. Right ventricular function can also be altered in patients with COPD and can be investigated with to modern diagnostic methods [46–49]. Treatment with *ß*_1_-receptor blockers, such as metoprolol, bisoprolol, and nebivolol (typically used in elderly patients), but also the less-prevalent *ß*-blocker carvedilol improves survival in HF and is recommended in patients with HF who also have COPD. Selective *ß*_1_-receptor blockers should be preferred to treat patients with COPD for approved cardiovascular indications and not for the purpose of preventing exacerbations of COPD [50]. Acute HF should be treated according to usual HF guidelines since there is no evidence to support an alternative management strategy. Non-invasive ventilation added to conventional therapy improves outcomes for patients with hypercapnic respiratory failure due to an exacerbation of COPD as well as those with HF and acute pulmonary edema [51].

### Ischemic heart disease (IHD)

Patients with COPD often suffer IHD. Different murine and clinical studies have shown that systemic inflammation and oxidative stress are important shared mechanisms between COPD and atherosclerosis, thus favoring IHD [52–54]. During, and for at least 90 days after ECOPD, there is an increased risk of cardiovascular events (e.g., deaths, myocardial infarction, stroke, unstable angina, and transient ischemic attack) in patients at a high risk of concomitant IHD [55]. Hospitalization for ECOPD has been associated with 90-days mortality of acute myocardial infarction, ischemic stroke, and intracranial hemorrhage [56]. Patients who demonstrate abnormal cardiac troponins during an ECOPD event are at increased risk of adverse outcomes including short-term (30-days) and long-term mortality [57, 58]. The treatment of IHD should be according to guidelines irrespective of the presence of COPD and vice versa.

### Arrhythmias

Cardiac arrhythmias are common in COPD and vice versa [59]. Atrial fibrillation is frequent and associated with a lower FEV_1_ [60]. In COPD patients presenting with severe worsening dyspnea, atrial fibrillation is frequently documented, and it may be either a trigger or a consequence of an ECOPD episode [61]. The presence of atrial fibrillation should not alter the treatment of COPD. Bronchodilators have been previously described as potentially pro-arrhythmic agents [62, 63]; however, available evidence suggests an overall acceptable safety profile for both LABAs and LAMAs [18]. Caution is advised, however, when using short-acting *β*_2_-agonists and theophylline, which may precipitate atrial fibrillation and make control of the ventricular response rate difficult [2]. Generally, theophylline should be avoided for the treatment of COPD as they do not significantly attenuate symptoms or reduce the risk of ECOPD [64].

### Peripheral vascular disease

Peripheral artery disease (PAD) is commonly associated with atherosclerotic heart disease and may have significant implications for functional activity as well as quality of life in patients with COPD [65]. In a large cohort of patients with COPD of all degrees of severity, 8.8% were diagnosed with PAD that was higher than the prevalence in non-COPD controls (1.8%) [65]. COPD patients with PAD reported a worse functional capacity and worse health status compared to those without PAD.

### Surgery in the CVD patient with COPD

Patients with CVDs often need surgery and/or percutaneous interventions. General surgical risk is increased in patients with COPD. The key risk factors include smoking, poor general health status, age, hypercapnia, obesity, and airflow limitation severity [2]. Common pulmonary postoperative complications include lung infections and atelectasis, which can lead to acute respiratory failure [2]. To prevent them, medical COPD treatment should be optimized before surgery. Specialist consultation is advisable. Comorbid conditions, particularly in patients with CVD, should be treated before any major surgical intervention.

## Conclusions

COPD is the elephant in the room for many patients with CVDs. It is a prevalent, preventable, and treatable condition, but most often it has not been diagnosed and, hence, not treated appropriately [2]. Importantly for cardiology practice, optimal management of COPD is associated with improved cardiovascular outcomes [66]. Yet, such clinical management requires, to begin with, a high level of suspicion and appropriate confirmation of diagnosis by spirometry. Initial treatment should focus on smoking abstinence and use of one or two long-acting bronchodilators. Referral to specialized pulmonary care is advised if response to treatment is not satisfactory.

## Data Availability

This article summarizes the main recommendations of the GOLD 2023 full document (in turn is based on 1594 references included there). This document can be accessed and freely downloaded from the GOLD website (www.goldcopd.org).
